# Effect of choline chloride supplementation on milk production and milk composition of Etawah grade goats

**DOI:** 10.1186/s40781-016-0113-5

**Published:** 2016-08-08

**Authors:** Supriyati, I. Gusti Made Budiarsana, Lisa Praharani, Rantan Krisnan, I. Ktut Sutama

**Affiliations:** Indonesian Research Institute for Animal Production, Bogor, 16720 Indonesia

**Keywords:** Choline chloride supplementation, Etawah Grade does, Forced drinking, Milk production, Milk composition

## Abstract

**Background:**

The effect of choline chloride supplementation through forced drinking combined with concentrate diets containing Ca-fish oil on milk production and milk composition of Etawah Grade goats was evaluated. Choline chloride is an essential component in ruminant diets as it is required for fat metabolism.

**Method:**

The experiment was conducted in a completely randomized block design with three types of treatments and eight replications. The trial had two successive experimental periods; the first, during the eight weeks of late pregnancy, and the second, during the first 12 weeks of lactation. Twenty-four Etawah Grade does in the second gestation period were divided into three treatment groups. Commercial choline chloride 60 % in corncobs-based powder was used as a source of choline chloride. The treatments were no supplementation (control) and supplemented with either 4 g or 8 g/2days of choline chloride. Choline chloride was given to the animals through a forced drinking technique, after dissolving it in 60 ml drinking water. The initial body weight of does was 38.81 ± 3.66 kg. The does were penned individually, and were given fresh chopped King Grass *ad libitum* and 700 g/day of concentrate diets containing Ca-fish oil, starting eight weeks prior to expecting kidding and continuing for 12 weeks of parturition.

**Results:**

All nutrient intakes were not significantly different (*p* > 0.05) among the treatments during the late pregnancy and the lactation periods. Supplementation did not affect (*p* > 0.05) the average daily gains and feed conversion ratio during pregnancy but gave effects (*p* < 0.05) on the average daily gains, feed conversion ratio and income over feed cost during lactation. The highest average daily milk yields and 4 % fat corrected milk yields were found in goats supplemented with 4 g/2days of choline chloride and increased by 17.00 % and 24.67 %, respectively, compared to the control. Moreover, milk composition percentage and milk constituent yields improved significantly (*p* < 0.05) in those supplemented with 4 g/2days of choline chloride.

**Conclusion:**

The supplementation of 4 g/2days of choline chloride through forced drinking increased milk yields, the 4 % fat corrected milk yields, milk composition, milk constituent yields, and improved feed conversion ratio and income over feed cost of Etawah Grade goats.

## Background

Choline is chemically known as β-hydroxyethyl tri-methyl ammonium hydroxide [1] and is an essential nutrient for normal animal growth and performance. Choline is a necessary component of cell walls, nerve transmission, fat metabolism and transport, and also an important source of labile methyl groups [2, 3]. Choline compounds cannot entirely be synthesized in the body; therefore, it is necessary to supplement choline in the feed, especially during the transition period. Choline can be absorbed from the lumen of the small intestine. Choline is available in the market as a mixture of choline chloride and wheat pollard or corncobs-based powder. Naturally, choline is found in barley, corn, corn gluten meal, fish meal, soybean meal, cotton meal, and alfalfa hay. The level of choline in all the feedstuffs is less than 0.68 mg/g of dry matter and digestibility values vary from 80 to 84 % [4].

The dietary supplementation in feeds apparently was ineffective in increasing the milk yield, because of rapid degradation of choline in the rumen [5, 6]. To improve the utilization of choline in ruminants, the previous researchers carried out choline chloride supplementation by dissolving it into drinking water, then administering it through abomasal infusion [6, 7], and duodenal infusion [8]. The administration of choline chloride through drinking water can also reduce the degradation of the choline compound in the rumen [2].

In the last decades, the addition of choline chloride in feeds in a form protected from rumen degradation, namely rumen-protected choline (RPC), improved nutrient digestibility, feed efficiency, milk production and milk quality in dairy cows [3, 9] and dairy goats [10]. Choline chloride supplementation also improved feed efficiency and increased income over feed cost (IOFC) [8], increased the conception rate and the pregnancy rate in cows [11] and increased productive performance in goats [12]. However, this protected form of choline chloride is not available in the local market. As reported by Davidson et al. [13] that the Ca-salts of fatty acids was used to protect choline supplementation. Therefore, the present trial evaluated the effect of choline chloride supplementation through forced drinking combined with concentrate diets containing Ca-fish oil on milk production and milk composition of Etawah Grade goats.

## Methods

### Choline chloride supplementation

Commercial choline chloride 60 % in corncobs-based powder was used as a source of choline chloride. Choline chloride was administrated through a forced drinking technique by which choline chloride 4 g or 8 g was dissolved in 60 ml of drinking water and then the dissolved choline chloride was poured it into a 100 ml of plastic syringe. Prior to the administration of the dissolved choline chloride, the needle of the syringe was pulled out. Then the dissolution of choline chloride was inserted into the goat by opening the goat’s mouth and pushed the dissolution into its mouth. The administration of choline chloride was carried out every two days in the morning before feeding during the pregnancy. Then, the administration continued every two days in the morning after milking and before feeding during the twelve weeks of lactation period. Feeding the animals was carried out approximately one hour after providing the dissolved choline chloride. At this time, we proposed that the dissolved choline chloride in drinking water might flow completely through the rumen.

### Experimental animals and management

The experimental conditions and animal procedures were handled in agreement with guidelines described by the Small Ruminant Laboratory Program at the Indonesian Research Institute for Animal Production. Goats were free from any disease, with a normal healthy appearance, and were penned individually in the same shed. Uniform management was followed for all goats. Selection of does was based on parity and milk yields of the previous lactation.

The experiment was conducted in a randomized complete block design with three treatments and eight replications. The trial had two successive experimental periods; the first during the eight weeks of late pregnancy, and the second, the first 12 weeks of lactation. Twenty-four Etawah Grade goats in the second gestation period, with initial body weight of 38.81 ± 3.66 kg were randomly distributed into three treatment groups and assigned to the same experimental diet. The treatments were no supplementation (control), or supplemented with either 4 g or 8 g every two days (g/2days) of choline chloride. The does were given fresh chopped King Grass *ad libitum* and 700 g/day of concentrate diets starting eight weeks prior to expecting kidding and continuing for 12 weeks of parturition. The ingredient and nutrient composition of grass and the concentrate diets fed to goats are presented in Table 1. A similar amount of Ca-fish oils salts were included in both the control and choline chloride treatments to provide equal amounts of fat to all treatments. Grasses and concentrate diets were offered twice daily in two almost equal meals in the morning and afternoon. Drinking water was available anytime through the nipple. Total feed (grasses and concentrate diets) intake was measured daily, and the animals were weighed every two weeks before feeding during gestation period or after milking and before feeding during lactation period.

**Table 1:** Ingredients and chemical composition of grass and concentrate diet fed to goats (on dry matter basis)

	Percent	Percent
Ingredient		
Pollard	35.00	
Soybean meal	18.00	
Cassava waste	38.00	
Molasses	5.00	
Ca-fish oil	1.50	
Mineral and vitamin mixed	2.00	
Probiotic	0.50	
Nutrient composition	Grass	Concentrate diet
Crude protein	10.51	14.53
Crude Fat	0.80	4.55
Neutral detergent fiber	71.03	26.08
Acid detergent fiber	49.35	12.92
Gross energy (kcal/kg)	3871	4085
Total digestible nutrients^a^	66.48	70.16
Ash	9.89	12.41
Calcium	0.26	2.66
Phosphorus	0.18	0.55

^a^The total digestible nutrients (TDN) obtained by calculation according to Nutrient Requirements Council (1981). Metabolized energy (kcal/kg) =0.62 times Gross energy and the %TDN = Metabolized energy (kcal/kg) divided by 0.0361

Parameters estimated were nutrient intakes of dry matter (DM), crude protein (CP), gross energy (GE), crude fat (CF), neutral detergent fiber (NDF), acid detergent fiber (ADF), calcium (Ca), and phosphorus (P); body weight (BW) changes; average daily gain (ADG) of does; and feed conversion ratio (FCR). The FCR value during pregnancy was determined as the amount of DM intake divided by ADG of does.

### Chemical analysis

The DM, CP, CF, NDF, ADF, Ca and P contents were analyzed in the grass and concentrate diet according to AOAC [14], modified in our laboratories. The DM was determined by drying at 135°C for two hours (Method 930.15). The CP (nitrogen × 6.25) content was determined by micro-Kjeldahl digestion and auto-analysis procedure, using auto-analyzer Brand Luebe, Germany (Method 990.02). Extraction with a solvent determined the CF content (Method 920.39). Digestion with a neutral detergent solution (Method 2002.04), analyzed the NDF content. The ADF content was measured by digestion with an acid detergent solution (Method 973.18). An ashed sample was dissolved in acids (a mixture of HCL and HNO3), and then Ca and P were determined by using AAS Model Varian Spectra 220 (Method 968.08) and UV–VIS Spectrophotometer (Method 965.17), respectively. Gross energy (GE) value was determined by bomb calorimeter (Adiabatic Oxygen Bomb, Parr Instrument Co. 6400) using thermochemical benzoic acid as a standard. The GE values were used to calculate metabolized energy (ME). The ME (Kkal/kg) = 0.62 times GE (Kkal/kg) and the percentage of total digestible nutrients (%TDN) = ME (Kkal/kg) divided by 0.0361, as described by Nutrient Requirements Council (NRC) [15].

### Milk yields and samples

Goats were milked by hand in the morning and evening. Individual morning and evening milk yields were recorded daily for each goat. The 4 % fat corrected milk (FCM) for each goat was calculated from milk yields and the percentage of milk fat using the formula as given by Gains [16]. The four percentage of FCM = (0.4 x milk yield) + (0.15 x milk yield x % fat).

The FCR value during lactation was determined as the amount of DM intake required to produce 1 kg 4 % FCM yield. Income over feed cost (IOFC) was measured as the income from milk yields minus feed cost which was the price of grass, concentrate diets and choline chloride per kg that was U$.0.054, U$.0.39 and U$. 10.71, respectively, with milk sales price, was U$. 1.79 per litre.

Milk samples from the consecutive evening and morning milkings were collected from each goat on day seven at the first week of lactation. Approximately 30 ml of milk from each goat were composited and analyzed for milk compositions of fat, protein, lactose, solids non-fat (SNF) and specific gravity were analyzed using Lacto-Scan Milk Analyzer. Total solids (TS) composition was calculated by adding the fat plus SNF contents.

### Statistical analysis

Data were evaluated statistically by a standard analysis of variance using SAS program [17]. If there was a significant difference between treatments, the difference then was compared using Duncan’s Multiple Range Test.

## Results and discussion

### Technique of giving choline chloride

In our study, we preferred to use unprotected choline chloride to protected form of choline chloride, mainly due to availability of choline chloride in local market and its low cost. Choline chloride used in this study was commercial choline chloride 60 % in corncobs-based powder which was available in the market.

The mode of administration was important since the mode could influence the bioavailability of choline to the animal. The earliest administration method of unprotected choline supplementation was the inclusion in concentrate diet or top of feed [5, 9, 18] and its post-rumen infusion [7, 8]. These methods were effective in increasing milk yields and milk composition of dairy cows. However, the supplementation of choline chloride through inclusion in concentrate diets or on top of feed, only a little value could be used as supplement compound due to rapid degradation of choline in the rumen [5]. Furthermore, the method of post-rumen infusion was difficult to adopt due to the requirement of high skilled technician and the limitation of facilities for the post-rumen infusion. Therefore, we developed a force drinking method in the administration of choline chloride to dairy goat.

The forced drinking method was developed, based on the above consideration and the invention of previous inventor [2]. Sleurink [2] invented that a method of feeding a non-encapsulated choline compound to a ruminant animal through drinking water enabled most of choline compound to by-pass the rumen. This was due to the liquid feed flow through the rumen. The choline chloride in drinking water consumed by ruminants passed quickly through the rumen. Approximately 60 to 70 % of the chlorine chloride in drinking water bypassed the rumen. The exact percentage that bypassed to the rumen depended on the concentration of choline chloride in the drinking water, the volume of water consumed, and the rate of the water consumed. In this study, the forced drinking method was applied with the hope that a part or all of dissolved choline chloride would bypass to the rumen.

The method of the forced drinking comprised two-steps. The first step was to dissolve a choline chloride in drinking water, and the second step was to provide choline chloride-containing drinking water to the goats. Whereas, the first step was to dissolve 4 or 8 g of choline chloride dissolved in 60 ml of drinking water, and the second step was to administrate the dissolved choline chloride in drinking water by inserting it to the mouth of the goat. Prior to the inserting, the dissolved choline was transferred to un-needle the plastic syringe and let the dissolution pass through rumen. The goats offered feeds approximately one hour after administration of dissolved drinking choline. At that time it was assumed that most of the dissolved choline chloride enabled to bypass the rumen of goats.

The dose of choline chloride used as supplement based on the previous researchers [10, 18]. In an experiments conducted by Bonomi et al. [18] it was found that the oral administration of 10 g/day unprotected choline which was equal to with 2 g/day of RPC, improved milk yields of dairy cattle, meaning that the level of unprotected choline would be 1 g/day for supplementation in goat because a goat’s body weight is approximately tens times lighter than a cow’s body weight. Furthermore, Pinotti et al. [10] reported that feeding 4 g/day of RPC (25 % choline content) would be an optimal dose in lactating dairy goat. Donkin [19] estimated the minimum choline needed in dairy cows for maintenance function (based on metabolic size) was approximately 4 to 6 g/day. Thus, we decided to supplement unprotected choline chloride (60 % of choline chloride in corncob-based powder) at level of 4 or 8 g/2days to the goat, which was almost equal to or double doses of Bonomi’s usage. The force drinking method with the dose of 4 or 8 g/2days of choline chloride, it seemed that choline was adequate and effective for its supplementation in the goats indicated by the increase of milk production and milk constituents as shown in Table 4.

Recently advance in choline supplementation, that was, the RPC had been used due to higher choline availability compared to unprotected choline. The RPC could have a favorable effect on milk production [9, 10]. The RPC could be given by mixing it with concentrate diet, and then fed to dairy cows [9] and dairy goats [10]. Janovick Guretzky et al. [20] fed Holstein and Jersey cows diets that were top-dressed once daily with RPC product.

### Nutrient intake

The total feed (grass and concentrate diets) and nutrients intakes, and the percentage of grass to total feeds intakes were not significantly different (*p* > 0.05) among the treatments during the late pregnancy and the lactation periods (Table 2). The different levels between 4 g and 8 g/2days dissolved choline chloride in water gave no differences in nutrient intake during the pregnancy and lactation periods.

**Table 2:** Total feed and nutrient intake of goats supplemented with different levels of choline chloride through forced drinking during pregnancy and lactation periods^a^

Variables	Level of choline chloride^b^			SEM	*P* values
	g/2days				
	0	4	8		
Pregnancy period					
Grass dry matter, g	559	631	569	67.52	0.094
Total dry matter, g	1177	1248	1187	67.47	0.095
Grass/Total dry matter, %	47.43	50.36	47.72	2.93	0.112
Organic matter, g	1045	1110	1054	60.78	0.095
Crude protein, g	148	156	150	7.03	0.094
Total digestible nutrients, g	805	853	812	44.76	0.094
Neutral detergent fiber, g	558	609	565	47.78	0.091
Acid detergent fiber, g	356	391	361	33.30	0.092
Crude fat, g	34.83	35.69	34.97	0.81	0.098
Calcium, g	17.90	18.09	17.93	0.18	0.091
Phosphorus, g	4.40	4.53	4.30	0.24	0.176
Lactation period					
Grass dry matter, g	574	615	611	58.42	0.318
Total dry matter, g	1192	1233	1228	58.46	0.315
Grass/Total dry matter, %	47.97	49.83	49.62	2.36	0.250
Organic matter, g	1058	1096	1092	52.60	0.307
Crude protein, g	150	155	157	6.19	0.142
Total digestible nutrients, g	836	865	862	41.00	0.314
Neutral detergent fiber, g	569	598	595	41.52	0.317
Acid detergent fiber, g	364	385	383	29.02	0.320
Crude fat, g	35.00	35.50	35.47	0.70	0.286
Calcium, g	17.93	18.04	18.04	0.15	0.289
Phosphorus, g	4.42	4.51	4.50	0.11	0.227

^a^On a dry matter basis

^b^Basal diet was supplemented with choline chloride through forced drinking technique

*SEM* = standard error of the means

Choline chloride supplementation through forced drinking did not significantly affect the main daily DM, CP, TDN, Ca and P intakes of feed during late gestation (Table 2). The mean daily DM and TDN intakes during late pregnancy in this trial were in the range of Kearl’s recommendation [21] but less than the Nutrient Requirement Council (NRC) requirement [22]. According to Kearl [21] the requirements of DM and TDN intakes for the late pregnancy of dairy goats at 40 kg to 50 kg of BW and 120 g ADG were 1.21 to 1.43 kg and 0.84 to 1.00 kg, respectively. Meanwhile, the requirements of DM intake and TDN for dairy goats at 40 kg of BW during late pregnancy with a single kid and 106 g ADG were 1.33 kg and 0.88 kg, correspondingly [22]. The main daily intake of CP in this trial was in the range of NRC [15] requirement and Kearl’s recommendation [21]. The requirements of total protein for the late pregnancy of dairy goats at 40 to 50 kg of BW and 120 g ADG was 159 to 173 g [15] and 129 to 153 g [22], respectively. The main daily intakes of Ca and P in this trial were higher than the NRC [15] and Kearl [21] requirements. The requirements of Ca and P for the late pregnancy of dairy goats at 40 to 50 kg of BW and 120 g ADG were 2.8 to 3.5g and 3.5 to 4.2 g [15] and 4 to 5 g and 2.8 to 3.5 g [21], respectively. From the above results, the nutrient intake of goats during late pregnancy was adequate to meet the nutrient requirements as recommended by Kearl [21].

In this trial, choline chloride supplementation did not affect the DM intake during late pregnancy, as also reported by previous researchers [20, 23, 24]. They reported that supplementation of choline into diets during 21 days before calving through 63 days after calving of cows did not affect the DM intake.

Moreover, choline chloride supplementation through forced drinking did not significantly affect the main daily DM, CP, TDN, Ca and P intakes of feed during the lactation period (Table 2). The mean daily DM and TDN intakes during lactation in this trial were less than the requirements of DM and TDN intakes according to Kearl [21]. Kearl [21] recommended that the requirements of DM intake and TDN for lactating goats of 40 kg BW and −20 g ADG were 1.90 kg and 1.05 kg, respectively. Meanwhile, the requirements of DM and TDN intakes for dairy goats at 40 kg of BW at lactation with a single kid and 106 g ADG were 1.67 kg and 0.84 kg, correspondingly [22]. The intake of CP was in the range of NRC requirements [15] and Kearl’s recommendation [21]. The requirements of total protein for lactating goats at 40 kg of BW and −20 g ADG were 159 g [15] and 160 g [21], respectively. The intakes of Ca and P were higher than NRC requirements [15] and Kearl’s recommendation [21]. The requirements of Ca and P for lactating goats at 40 kg of BW and −20 g ADG were 3.5 g and 4.2 g [15] and 5 g and 3.5 g [21], respectively. From the above results, the CP, TDN, Ca, and P intakes of goats during lactation were adequate to meet the nutrient requirements as recommended by NRC.

During lactation period, choline chloride supplementation did not affect the DM intake, which was attributed to no increase of other nutrient intakes. Previous studies [23, 25] reported the same results that increasing dietary choline had no effect on DM intake of lactating dairy cows. However, our findings were in contrary to those obtained by other researchers [9, 26]. Mohsen et al. [9] found that RPC supplementation increased the TDN intake. Furthermore, Zahra et al. [26] reported that the supplementation of protected choline improved DM intake of cows during the last three weeks of pre-partum through four weeks of postpartum. The variation of DM intakes in the referred research works might be due to the difference between the choline forms used. In this trial choline chloride was used as rumen unprotected. Furthermore, the difference in the DM intakes might also due to animal differences, differences in the nutritive value of the diets being fed, level of choline intake, the administration of choline and experimental design.

The choline chloride supplementation had not influenced (*p* > 0.05) the percentage of grass intake to total feed intakes (Table 2), indicating that the ratio of forage to concentrate in this trial would not influence the milk production. As reported by Tufarelli et al. [27] that the ratio 35/65 forage to concentrate provides greater milk production compared to 50/50 ratio and 65/35 ratios without influencing the milk composition during lactation period of Jonica breed goats.

### Body weight, ADG and FCR performance of goats

The supplementation with choline chloride did not affect (*p* > 0.05) BW pre-parturition, ADG and FCR values during the last eight weeks of pregnancy (Table 3). There were not different effects on BW, ADG and FCR during pre-partum period due to the same nutrients intake of goats among the treatments. These results were in agreement with those obtained by earlier researchers [9, 20, 25] who supplemented cows’ diets with choline during the late pregnancy. Pinotti et al. [10] reported that the RPC supplementation in per-parturient dairy goats had no effect on BW change during pregnancy. Furthermore, Elek et al. [28] reported that supplementation of RPC did not affect the body condition score of cows during the last three weeks of pregnancy. However, Pinotti et al. [29] found that choline supplement enhanced growth performance of finishing cattle, even though the mechanism by which choline induced this effect was unknown. A possible explanation could be the fact that the animals used in their experiment were in adaptation period.

**Table 3:** Body weight, average daily gain and feed conversion ratio of goats supplemented with different levels of choline chloride through forced drinking during pre-parturition and parturition

Variables	Level of choline chloride^a^ (g/2days)			SEM	*P*-value
	0	4	8		
Pre-parturition					
Initial body weight, kg	38.88	38.75	38.81	3.81	0.998
Body weight pre-parturition, kg	45.38	45.25	44.83	4.02	0.958
Average daily gain during late pregnancy, g	116.07	119.07	107.14	33.18	0.826
Feed conversion ratio, DMI/ADG	10.14	10.48	11.08	2.85	0.840
Parturition					
Body weight at kidding, kg	36.75	37.13	35.80	3.64	0.757
Body weight decreased at kidding, kg	8.63	8.13	9.01	1.69	0.584
Body weight at 12 weeks lactation, kg	37.38	40.44	40.06	3.24	0.144
Average daily gain during the first 12 weeks lactation, g	7.44^c^	39.44^b^	50.74^b^	23.08	0.003

^a^Basal diet was supplemented with choline chloride through forced drinking technique

^b,c^Values followed by different superscripts in the same row differ significantly (*p* < 0.05)

*DMI/ADG* dry matter intake/average daily gains

*SEM* standard error of the means

Supplementation of choline chloride did no influence (*p* > 0.05) of BW at kiddings, the BW decreased at kidding and the BW at the first 12 weeks of lactation but increased ADG (*p* < 0.05) during the first 12 weeks of the lactation period (Table 3). The different levels of 4 g and 8 g/2days of dissolved choline chloride in water gave no differences in the ADG during the lactation period. All does did not suffer the loss of weight during the lactation period. Our results were in agreement with those obtained by earlier researchers [30] who supplemented cows’ diets with choline during the first five weeks of lactation, improved the change of body condition score compared to control goats. Similarly, Pinotti et al. [10] reported that choline-supplemental goats recovered BW more quickly in weeks 3 and 4. The treated goats increased ADG than the control does in the trial, indicating that the does supplemented with choline chloride mobilized less body tissues. It is still unclear which mechanism involved in the increase of ADG during lactation affected by supplementation. However, it might be due to the alterations in lipid metabolism and/or transport whereas blood free fatty acids (FFA) increased, suggesting that choline may have an effect on transportation and mobilization of FFA from adipose tissue, and a possible role of choline in milk fat deposition [23]. Furthermore, the administration of choline carried out before and after parturition may be particularly beneficial this time in view of adipose tissue and liver metabolism changes that occur during transition from late pregnancy to early lactation [24]. Moreover, choline produces betaine which involves the influence of performance of amino acids, protein and energy metabolism which could be attributed to a better production performance [3]. Leiva et al. [31] found that supplementing RPC to transition dairy cows enhanced the serum heptaglobin response, increased serum insulin concentration or enhanced per-parturient acute-phase protein response, and was one of the mechanisms by which RPC supplement benefits health and production parameters of transition dairy cows.

However, the results of our study disagreed with those obtained by others [7, 24, 30] who supplemented cows’ diets with choline which had no improvement of ADG during the lactation. The differences of our results from those ones might be due to the presence of Ca-fish oil and choline chloride in the diets in this trial. In this study, the Ca-fish oil was included in the formulation of concentrate diets in an attempt to improve the energy status of dairy goats during the per-parturient period and to improve milk conjugated linoleic acids (CLA) content. Another benefit of providing Ca-fish oil in the diet increased the dietary fat as long chain fatty acids (LCFA) intake, which might help to decrease concentrations of non-esterified fatty acids (NEFA) and to prevent occurrence of ketosis. Dietary LCFA are absorbed into the lymphatic system and do not pass first through the liver. This fat can provide energy for peripheral tissue and the mammary gland. The increased energy availability would, in turn, decrease mobilization of body fat and NEFA concentration [32]. Moreover, in this study we predicted that Ca-fish oil blended with choline chloride in digestion system of a goat to form protected form of choline chloride. As reported by Davidson et al. [13] that the rumen protected form of choline chloride was produced by the processing of choline chloride and Ca-fish oil.

### Milk production

Table 4 shows the effect of choline chloride supplementations on an average daily milk yield, a 4 % FCM yield, total milk yields for 12 weeks of production, FCR, IOFC, milk composition, and constituent milk yields.

**Table 4:** Effect of choline chloride supplementation through forced drinking on milk yields, milk composition and milk constituent yields responses of goats

Variables	Level of choline chloride (g/2days)^a^			SEM	*P* values
	0	4	8		
Average daily milk yields, ml/d	659^d^	771^c^	697^cd^	77.34	0.039
Average daily milk yields, g/d	678^d^	793^c^	717^cd^	77.34	0.039
Average daily 4 % FCM yields, g/d	756^d^	943^c^	870^cd^	91.28	0.004
Total milk yields 12 weeks, kg	56.90^d^	66.54^c^	60.18^cd^	6.53	0.034
Feed conversion ratio^b^	1.58^c^	1.31^c^	1.41^d^	0.07	<0.0001
Milk composition, %					
Fat	4.77^c^	5.26^d^	5.42^c^	0.07	<0.0001
Protein	3.49^d^	3.61^c^	3.65^c^	0.10	0.012
Lactose	6.23^d^	6.28^d^	6.56^c^	0.09	<0.0001
Specific gravity	1.028	1.028	1.028	0.00	0.251
Solid non fat	10.19^d^	10.47^c^	10.45^cd^	0.09	0.009
Total solids	15.24^d^	15.45^cd^	15.87^c^	0.45	0.036
Milk constituents yields, g/d					
Fat	32.29^d^	41.71^c^	38.86^c^	4.02	0.001
Protein	23.63^d^	28.63^c^	26.17^cd^	2.78	0.013
Lactose	42.18^d^	49.77^c^	47.00^cd^	6.46	0.039
Solid non fat	70.93^d^	80.76^c^	74.88^c^	10.63	0.009
Total solids	103.24^d^	122.45^c^	113.71^cd^	11.97	0.038
Income over feed cost, U$	0.93^d^	1.11^c^	0.97^cd^	1.883	0.037

^a^Basal diet was supplemented with choline chloride through forced drinking technique

^b^Feed conversion ratio = dry matter intake/4 % fat corrected milk yields

^c,d^Values followed by different superscripts in the same row differ significantly (*p* < 0.05)

*SEM* standard error of the means

4 % fat corrected milk (FCM) = (0.4 x milk yields) + (0.15 x milk yields x % fat)

**Fig. 1:**
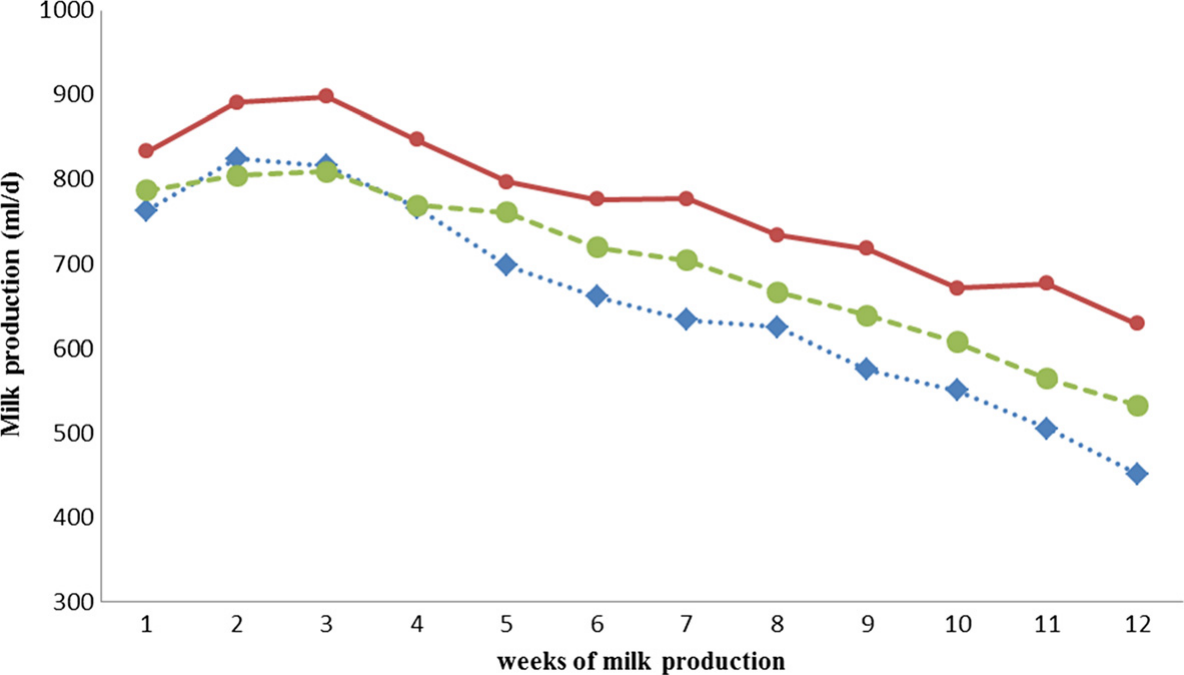
The daily average milk production of goats supplemented with choline chloride throgh drinking technique during 12 weeks lactation periods. . CC = supplemented with choline chloride every two days. From weeks 1 to 4, there was no significant difference (*p* > 0.05) in daily milk production between non-supplemented and supplemented at both levels, but afterwards, there was a significantly difference (*p* < 0.05) in milk production between non-supplemented and supplemented with 4 g/2days of choline chloride. From weeks 5 to 12, there was no significantly difference (*p* > 0.05) in milk production of goats between non-supplemented and supplemented with 8 g/2days

Supplementation of 4 g/2days of choline chloride increased average daily milk yields, average daily 4 % FCM yields and total milk yields for 12 weeks significantly (*p* < 0.05). The highest average daily milk production was found for goats supplemented with 4 g/2days of choline chloride, followed by goats supplemented with 8 g/2days of choline chloride and then the control ones (Fig. 1). From weeks 1 to 4, there was no significant difference (*p* > 0.05) in daily milk production between non-supplemented and supplemented at both levels, but afterwards, there was a significant difference (*p* < 0.05) in milk production between non-supplemented and supplemented with 4 g/2days of choline chloride.

In this trial, using a forced drinking technique to supplement choline chloride, the milk yields increased by 17.00 % and 5.77 %; the 4 % FCM yields improved by 24.67 % and 15.00 %; and milk fat yields increased by 29.17 % and 20.35 %, respectively, for the goats supplemented with 4 and 8 g/2days of choline chloride compared to the control. But no differences for milk and FCM yields between control and supplemented goats at the level of 8 g/2days of choline chloride.

The increases in milk yields and FCM yields in this trial were similar to the results reported by Erdman et al. [23]. They found that a supplement of 4 g/d choline to the basal diet of 75 % concentrate and 25 % corn silage improved milk yields and FCM yields of dairy cows by 12.7 % and 28.2 %, respectively, compared to the control. Sharma and Erdman [8] reported that infusion of 30 g choline into the abomasums of dairy cows was effective in increasing milk and fat yields. Similarly, Pinotti et al. [10] reported that the supplementation 4 g /day of RPC in Saanen goats, the milk yields and 4 % FCM yields were, respectively, 210 and 350 g/day higher in RPC supplemented goats than the non-supplemented goats. However, Atkins et al. [5] found that dietary supplementation apparently was ineffective due to rapid degradation of choline in the rumen. The results of our trial indicated that supplementation of choline through forced drinking was effective in increasing milk yields and milk fat production. Whereas, choline has two functions, that is, to supply methyl donor betaine and can be used as choline, primarily as a component of phospholipids. Choline is required for lipid and cholesterol transport and metabolism and also an essential component of very low density of lipoproteins (VLDL) [33]. The mechanism by which choline improved the milk production and FCM yields might due to the alterations on lipid metabolism and/or transport [23], its effect on liver lipid metabolism [33] and the elevating the export of triglycerides from the liver and by sparing methionine as a methyl donor [34].

The improvements of milk yields and fat yields might also be due to the presence of Ca-fish oil in concentrate diets. In one possible mechanism, Ca-fish oil might be interacted or blended with choline chloride in digestion system of a goat to form protected of choline chloride. As reported by Davidson et al. [13] that calcium salts of fatty acids were used to protect choline supplements as RPC, which gave greater availability of choline, improved milk production in goats. Furthermore, the Ca-fish oil supply in the feed could improve milk fatty acids profile and eventually the health of the goats [12] resulted in increasing milk yields, fat yields and BW.

There were no differences for milk and FCM yields between control and supplemented goats at the level of 8 g/2days of choline chloride. With the increasing level of choline, the milk yields response decreased. This result was similar as reported by previous studies [23, 24]. Erdman et al. [23] reported that the level of 4 g/d choline gave better response on the milk yields and milk composition of cows compared to the level 2 or 6 g/d choline. Piepenbrink & Overton [24] reported that the highest milk and FCM yields obtained from the cows supplemented with 45 g/d of RPC (contained 25 % choline chloride) compared to cows supplemented with 60 and 75 g/d of RPC which were similar to cows control. From the above results and the referred research works indicated that there was a quadratic tendentious response of choline supplementation on milk and FCM yields. The possible explanations why no differences on milk and FCM yields between control and higher level (8 g/2days) of choline chloride supplementation in this trial might due to the fact that the high level of choline might affect the rumen pH [6, 23] and milk urea N concentration [24]. Although this trial lacked data about rumen pH and milk urea N concentration of goats, but as reported by the previous results [6, 23, 24] that higher choline decreased the rumen pH [6, 23] and milk urea N concentration [24] due to its chemical high reactivity of choline. When rumen pH decreased, the protozoa population tended to decline, which it was possible that the flow of choline to duodenum for absorption was also reduced. A low value of rumen pH would also affect the rumen digestibility which was attributed to the performance of goats. Furthermore, Sharma & Erdman [6] reported that the higher choline supplementation which reduced rumen pH with no plausible explanation could be made for lower pH associated with highest choline supplementation. Milk urea N concentrations decreased linearly as cows consumed increasing amounts of RPC, this response could be attributed to changes in milk yields rather than a direct effect of RPC [24].

Choline chloride supplementations improved FCR (DMI/4%FCM yield) very significantly (*p* < 0.0001) by 17.09 % and 10.76 % for goats supplemented with both 4 and 8 g/2days of choline chloride, respectively. The supplementations at 4 g/2days of choline chloride also increased IOFC by 18.28 % compared to the control. These results agreed with those obtained by Mohsen et al. [9] who showed that the cows fed RPC had improved the FCR values and economic efficiency. But Rahmani et al. [30] reported that supplementation of RPC into the diets of early lactating dairy cows did not affect feed efficiency. This improvement of FCR and IOFC in this trial, might be due to the improvement of milk and 4 % FCM yields.

### Milk composition and milk constituent yields

Table 4 shows the milk composition and milk constituent yields. Choline chloride supplementation increased milk composition of fat and lactose (*p* < 0.0001), protein, SNF and TS (*p* < 0.05), but the supplementation did not influence the specific gravity. The milk composition in dairy animals was directly impacted by nutrient intake [31], whereas the nutrient intakes were similar between treatments in this trial. Hence, choline supplementation impacted the milk concentration of fat, protein, lactose, SNF, and TS despite similar nutrient intake between treatments.

The different choline chloride levels did not affect the milk composition except on fat contents (*p* < 0.0001). Milk fat and TS of goats in this trial were in the range reported by Sutama [36] for Etawah Grade goats in the tropical region, from 4.42 to 6.4 %, and 13.62 to 15.72 %, respectively. Milk protein and milk lactose of EG goats in this trial were different from the values reported by Sutama [35]. He also reported that milk protein and milk lactose of Etawah Grade goats were 3.78 to 4.52 % and 5.08 to 5.62 %, respectively. Furthermore, Mulyati et al. [36] reported that milk fat of Etawah Grade goats fed Elephant grass, gliricidia leaves and the concentrate was higher (6.1 to 6.64 %) than our results.

Milk constituent yields increased significantly (*p* < 0.05) in supplemented goats at the level of 4 g/2days of choline chloride compared to control goats (Table 4). The milk of fat, protein, lactose and SNF yields increased by 29.17 %, 21.16 %, 17.99 % and 13.86 %, respectively, in supplemented goats at the level of 4 g/2days of choline chloride compared to the control goats. There were no differences for milk protein, lactose and TS yields between control and supplemented goats at the level of 8 g/2days of choline chloride. The reason why there were no different protein, lactose and TS yields between control and 8 g/2days of choline chloride supplementation might be due to the consequence on the differences of milk yields at the both levels (0 and 8 g/2days) of choline chloride supplementation, reflecting no differences in milk protein, lactose and TS yields. Since, one of the choline actions was mainly for the alterations on lipid metabolism and/or transport [23], the different levels of choline influenced only fat and SNF yields.

Milk fat content was linearly increased with the increase of choline supplementation level indicated that the supplementation of choline gave more response in milk fat content than another milk composition due to better fat metabolism. The increases of milk fat and fat yields in this trial were in agreement with those obtained by Erdman et al. [23]. They reported that a supplement of 4 g/kg choline in concentrate improved milk fat compared to the control. Other researchers [9, 24, 30] also reported that feeding RPC improved the milk fat of cows. Similarly, Pinotti et al. [11] reported that supplementation of 4 g/day RPC improved the milk fat of goats from 3.68 to 3.98 % and the fat yields improved 20 %. The increase of fat yields in this trial was as a consequence of higher milk yields and milk fat. In this mechanism, the increase of fat yields might be due to the presence of choline contributing to fatty acid transport of mobilized of free fatty acid from adipose tissue through mammary gland, and it might enhance the availability of fatty acid for milk fat synthesis [3, 23].

Milk protein and milk protein yields in this trial also increased significantly for goats supplemented with 4 g/2days of choline, which was in agreement with Erdman & Sharma [25] and Hartwell et al. [37] who reported that feeding choline improved milk protein of cows. The increase of milk protein yield was in line with Pinotti et al. [10], who reported that feeding RPC improved milk protein yields of goats by 12.62 %. The increase of milk protein yields of milk goats supplemented with 4 g/2days of choline chloride might be due to the presence of choline improved methyl group metabolism and allowed more methionine to be available for protein synthesis in the mammary gland or potential ability of choline to spare methionine [3, 10, 34]. Whereas, choline serves as methyl donor required for methyl group metabolism in which methyl donation is needed for an energy metabolism and protein synthesis especially during the transition period. With regard to dose in this trial, providing choline chloride 4 g/2days, a methyl group metabolism was sufficiently improved due to the time length of choline supplementation which carried out since eight weeks of expecting kidding and continued until weeks of lactation period. Particularly, with respect to a lactation cycle several studies provided convincing evidence that RPC positively affected the lactation performance especially when feeding was initiated prior to calving, and was continued during early lactation [24]. Furthermore, other researchers suggested the dose of choline supplementation [10, 18, 38] be variable depended on the animal and diets used, state of lactation and the mode of providing choline. Donkin [19] estimated the minimum choline needed in dairy cows for maintenance functions was approximately 4 to 6 g/day. Moreover, production responses were obtained when higher doses were fed [25].

Based on the above data, the increase of milk fat, fat yields, milk protein and protein yields in this trial indicated that the administration of choline through drinking method was effective. The link between choline supplementation and milk response has been attributed to the metabolic interchangeability of choline and methionine (originated from protein supply in concentrate diets), in the sense that both could furnish labile methyl groups [12].

Milk lactose composition increased significantly for goats supplemented with 8 g/2days of choline than the control, and 4 g/2days supplementation. Lactose yields increased significantly only for goats supplemented with 4 g/2days of choline. The differences in lactose yields for supplemented with 4 g/2days of choline than control and 8 g/2days supplementation due to more milk yields produced at level of 4 g/2days compared to other treatments. These results were disagreed with the results obtained by Zahra et al. [26] and Rahmani et al. [30] who showed that the cows fed RPC had no increase in milk lactose. Choline supplementation has been positively associated with milk concentration of lactose, whereas lactose is sources of milk energy. The increase of milk lactose concentration and lactose yields might be better in energy metabolism, since choline influenced in the increase of energy metabolism [3].

Milk SNF concentrations and SNF yields increased significantly, this trial disagreed with the results obtained by Zahra et al. [26] and Rahmani et al. [30] who showed that the cows fed RPC had no increase in milk SNF. Furthermore, milk TS (fat and SNF) concentrations and TS yields increased significantly. The increase of milk TS yields was in agreement with Mohsen et al. [9] who reported that supplementation of RPC improved TS of milk cows. Hence, the increase in milk TS concentration of choline chloride supplementation in goats detected herein should be attributed to the treatment effect detected for milk fat and SNF concentrations. The increase of milk TS yields in our study agree with those obtained by others [24] who showed that the cows fed RPC had increased TS contents and yields.

However, Rahmani et al. [30] reported that supplementation of RPC into the diets of early lactating dairy cows did not affect milk yields and milk composition. The differences in milk yields and milk composition with our results might be due to many factors such as basal diet composition, the dose and mode of administration of choline, forage to concentrate ratio [27], breed, traits and stage of lactation [39].

## Conclusion

Choline chloride supplementation through forced drinking water combined with concentrate diets containing Ca-fish oil was effective as a tool for supplementation into goats. Supplementation of choline chloride increased milk composition and milk constituent yields. The milk yields increased by 17.00 % and 5.77 %, the 4 % FCM yields improved by 24.67 % and 15.00 %, and milk fat yields increased by 29.17 % and 20.35 %, respectively, for the goats supplemented with 4 and 8 g/2days of choline chloride compared to the control. The IOFC increased by 18.28 % for the goats supplemented with 4 g/2days of choline chloride compared to the control. Further studies are required to compare choline chloride supplementation using the forced drinking water and concentrate containing Ca-fish oil technique with other techniques of supplementation which is easier and less labours or other form of choline (RPC) supplementation in lactating dairy goats.

## Abbreviations

ADG, average daily gain; AOAC, Association of Official Analytical Chemists; BW, body weight; Ca, calcium; Ca-fish oil, calcium-fish oil; CF, crude fat; CLA, conjugated linoleic acids; CP, crude protein; DM, dry matter; FCM, fat corrected milk; FCR, feed conversion ratio; GE, gross energy; HCL, hydrochloric acid; HNO3, nitric acid; IOFC, income over feed cost; LCFA, long chain fatty acids; NDF, neutral detergent fiber; NEFA, non-esterified fatty acids; NRC, nutrient requirement council; P, phosphorus; RPC, rumen protected choline; SAS, statistical analysis system; SEM, standard error of the mean; SNF, solids non-fat; TDN, total digestible nutrients; TS, total solids; UV–VIS, ultraviolet – visible
